# The Association between DNA Copy Number Aberrations at Chromosome 5q22 and Gastric Cancer

**DOI:** 10.1371/journal.pone.0106624

**Published:** 2014-09-11

**Authors:** Pei-Chien Tsai, Szu-Wei Huang, Hsiang-Lin Tsai, Cheng-Jen Ma, Ming-Feng Hou, I-Ping Yang, Yung-Song Wang, Suh-Hang Hank Juo, Jaw-Yuan Wang

**Affiliations:** 1 Hepatobiliary Division, Department of Internal Medicine, Kaohsiung Medical University Hospital, Kaohsiung, Taiwan; 2 Department of Medical Genetics, College of Medicine, Kaohsiung Medical University, Kaohsiung, Taiwan; 3 Department of Medical Research, Kaohsiung Medical University Hospital, Kaohsiung, Taiwan; 4 Graduate Institute of Medicine, Kaohsiung Medical University, Kaohsiung, Taiwan; 5 Graduate Institute of Clinical Medicine, Kaohsiung Medical University, Kaohsiung, Taiwan; 6 Cancer Center, Kaohsiung Medical University Hospital, Kaohsiung, Taiwan; 7 Division of Gastrointestinal and General Surgery, Department of Surgery, Kaohsiung Medical University Hospital, Kaohsiung, Taiwan; 8 Department of Nursing, Shu-Zen College of Medicine and Management, Kaohsiung, Taiwan; Duke Cancer Institute, United States of America

## Abstract

**Background:**

Gastric cancer is common cancer. Discovering novel genetic biomarkers might help to identify high-risk individuals. Copy number variation (CNV) has recently been shown to influence risk for several cancers. The aim of the present study was sought to test the association between copy number at a variant region and GC.

**Methods:**

A total of 110 gastric cancer patients and 325 healthy volunteers were enrolled in this study. We searched for a CNV and found a CNV (Variation 7468) containing part of the *APC* gene, the *SRP19* gene and the *REEP5* gene. We chose four probes targeting at *APC-intron8*, *APC-exon9*, *SRP19* and *REEP5* to interrogate this CNV. Specific Taqman probes labeled by different reporter fluorophores were used in a real-time PCR platform to obtain copy number. Both the original non-integer data and transformed integer data on copy number were used for analyses.

**Results:**

Gastric caner patients had a lower non-integer copy number than controls for the *APC-exon9* probe (Adjusted p = 0.026) and *SRP19* probe (Adjusted p = 0.002). The analysis of integer copy number yielded a similar pattern although less significant (Adjusted p = 0.07 for *APC-exon9* probe and Adjusted p = 0.02 for *SRP19* probe).

**Conclusions:**

Losses of a CNV at 5q22, especially in the DNA region surrounding *APC-exon 9*, may be associated with a higher risk of gastric cancer.

## Introduction

Gastric cancer (GC) is the fourth most common cancer and the third leading cause of cancer deaths worldwide in men; the fifth most common cancer and the fifth leading cause of cancer death in women [1]. According to the International Agency for Research on Cancer (IARC), Japan, China and Korea have a higher incidence rates of GC [2]. In Taiwan, GC was the sixth major cause of cancer-related death in 2010 (http://www.doh.gov.tw/statistic/index.htm; accessed in June 2011). Gastric cancer is highly complex and exhibits heterogeneity in clinical, biological, and genetic aspects. Known environmental factors that influence GC include *Helicobacter pylori (H. pylori)* infection, dietary habits, cigarette smoking, family history, and sex (a higher male-to-female ratio) [3]. As family history is a major risk factor for GC, recent studies have focused on the genetic factors that play a role in GC. Several investigators have documented the genetic alterations that are involved in the development of GC [4].

Gastric cancer often exhibits late clinical presentation, and it is usually diagnosed in the advanced stage and carries a poor prognosis. Early detection of GC is crucial for improving therapeutic efficacy, and reducing mortality, therefore, identifying relevant genetic biomarkers might help in the early detection of GC. A large number of associations between structural genomic changes and diseases susceptibility have been unraveled [5], [6]. Several specific genetic changes including duplication and mutation have been suspected or proven to be related to GC progression [7]. DNA copy number variations (CNVs) are common in several cancers and other disease endpoints. Variations in DNA copy number might be an indicator of high risk of GC in individuals. Using comparative genomic hybridization (CGH)/array-CGH (aCGH) analysis, several genomic regions have been found in GC cells or GC patients to have gains of DNA regions including 3q26–28, 7p12–15, 7q21–22, 8q21–24, 13q21–23, 17q21–22, 20p12, and 20q11–13 and losses of DNA regions including 4q26–27, 5q14–22, 9p21–23, 17p12–13, and 18q22 [8]–[13]. These results indicate that the patterns of chromosomal instability may correlate with the clinic-pathological characteristics of GC.

Previous studies have documented abnormalities in the adenomatous polyposis coli (*APC*) gene at chromosome 5q22 to result in familial adenomatous polyposis (FAP), hereditary non-polyposis colon cancer and other cancers [14]–[16]. Most frequent losses of the copy number at 5q22 in GC patients of difference racial are summarized in Table S1 in File S1 [8]–[10], [12], [13], [17]–[22]. Studies reported that 15.4% in Japanese [10], 35% in Korean [21], and 21% in Turk [20] of GC patients had gene mutations at 5q14–22. Losses of copy number at chromosome 5q22 has been found to be significantly associated with histological type [13], [18], [19], lymph node status [12] and metastasis [12] in GC patients. In addition to the relationship with gastric cancer, 5q loss was also often involved in premalignant stage [17], [22]. These studies have indicated that the *APC* gene may play a significant role in GC. Therefore, in this study, we tested the association of copy number at 5q22 with GC in the Taiwanese population.

## Methods

### Study population

A total of 110 GC patients and 325 healthy controls were enrolled from Kaohsiung Medical University Hospital in Taiwan. All patients were either Taiwanese or mainland Chinese. The presence of GC was also pathologically confirmed. The histologic grade was classified according to the criteria of Lauren [23]. The tumor staging was in accordance with the American Joint Committee on Cancer (AJCC) staging system [24]. Subjects with any other malignancies were excluded from the study. The control subjects were healthy volunteers who participated in regular health checkups at the same hospital. None of the controls had personal history of cancer or any other diagnosed significant gastric disorders at the time of enrollment. The study protocols and methods were approved by the Institutional Review Board of Kaohsiung Medical University Hospital. All participants provided written informed consent prior to the commencement of study.

### Select candidate CNV of GC-related

The candidate CNVs encompassing the *APC* gene at chromosome 5q22 were retrieved from a public database (database of genomic variants, DGV, http://projects.tcag.ca/variation). Until November 2010, the database listed physical positions for 66,741 CNVs located in 15,963 common CNV regions. Among them was a CNV (Variation 7468) at 5q22 that span 127.5 kb (chromosome location: 112,138,707 to 112,266,194, based on NCBI build 36/hg18 version) covering a part of the *APC* gene and *SRP19* gene at the forward DNA strand and the *REEP5* gene at the reverse DNA strand. Based on the array CGH data from 50 healthy French men, the frequency of gain and loss of copies at this region were 2% and 2%, respectively [25]. Literature shows six mutations in the alternatively-spliced region of exon 9 of the *APC* gene to be associated with FAP [26] and colon cancer [27], but none has been reported in relation to GC.

We chose two neighboring probes interrogating intron8 and exon9 of the *APC* gene, respectively, one probe for the *SRP19* gene, and one probe for the *REEP5* gene to detect the copy number of this CNV whereas *RPPH1* was used as a reference gene. These probes are commercially available from TaqMan (Applied Biosystems Inc (ABI), CA, USA) and their detailed information on genomes (build 36/hg18) is shown in **Table S2 in File S1** and **Figure S1 in File S1**.

### Genomic DNA preparation and real-time PCR for copy number detection

DNA isolation was performed using commercially available DNA isolation kits (QIAamp DNA mini kit, Qiagen, Hamburg, Germany). RNase A (Qiagen) was used to digest single-strand RNA for the isolation of RNA-free DNA. Genomic DNA was extracted from peripheral blood leukocytes. DNA was quantified first by UV absorption (Beckman DU 640 Spectrophotometer; Beckman Coulter, Brea, CA, USA) and then amplified by Real-Time PCR. DNA concentrations were adjusted to 10 ng/µl before genotyping. Real-Time PCR was performed using the Taqman probes in an ABI 7900HT Real-Time PCR instrument (ABI). Commercially available FAM dye-labeled probes were designed to amplify the *APC*, *SRP19* and *REEP5*. VIC dye-labeled *ribonuclease P RNA component H1 (RPPH1)* was used as the endogenous control because *RPPH1* has exactly two copies per diploid human genome, which is located on chromosome 14q11.2 [28]. Primers and probes were designed from genomic sequence (build 36/hg18) using the ABI proprietary software. The TaqMan copy number assay contained 1 µl *APC, SRP19 or REEP5* probe (20x, FAM labeled), 1 µl *RPPH1* probe mix (20x, VIC labeled), 10 µl TaqMan Universal PCR Master Mix (2x), 1.5 µl genomic DNA and 6.5 µl of water. The amplification protocol used for the reaction is 95°C for 10 min, followed by 95°C for 15 sec and 60°C for 1 min for 40 cycles. A manual threshold cycle threshold (Ct) of 0.2 and an automatic baseline were used to detect the template quantity of target genes and *RPPH1* gene by a sequence detection system software (ABI, version 2.4). For each sample, four probes (*APC-intron8, APC-exon9, SRP19,* and *REEP5*) were performed along with an internal control. The target probes and internal control were loaded at the same well and each reaction was performed in quadruplicates. CopyCaller software (ABI, version 1.0) was used to calculate the integer copy number of each probe based on the real-time PCR data. We calculated the mean and standard deviation (SD) of quadruplicates of ΔCt for each subject. To control for data quality, the data was filtered using three steps. Only the subjects who passed all three steps of data quality control were used in the subsequent analyses.

### Copy number quality control

For quality control of the data, the copy number of each probe from real-time PCR was filtered by three steps. In the first step, the data from individual real-time PCR runs were examined. The following criteria was applied for excluding for analysis: 1) VIC Ct>32, possibly due to a failure to amplify the internal *RPPH1* signal, 2) any probe with ΔCt>4.0 or 3) FAM Ct>40. Data that met with the latter two criteria suggested the failure of amplification of target probes, and therefore the data were considered as unreliable. After the first step, we calculated the mean ΔCt for each study subject.

The second step was to exclude the outlier of mean ΔCt using ±3 SDs as cutoffs. After the first and second steps, the copy number of each probe for each individual was calculated by the formula 2^−ΔΔCt^×2. Accordingly, the copy number may not be an integer. Given that the copy number is theoretically an integer, we further followed the guidelines of CopyCaller software to estimate the integer of each copy number using automatic maximum likelihood analysis method, based on the probability density distribution across all samples.

Finally, according to the distribution of the integer copy number, a standardized z score and the confidence value were calculated. A higher absolute value for the standardized z score and a lower confidence value implied greater variation. As suggested by the user’s guidelines of CopyCaller software (ABI, version 1.0), the third step for data quality control is to exclude any samples that met with both of the following criteria: 1) the absolute value of the z score >2.65 and 2) the confidence value <0.9. Only the participants who passed all three steps of the data quality control were used in the subsequent analyses.

### Statistical Analysis

Because only a few of the participants had a copy number of greater than 3 or less than one, the copy number was categorized into three groups (≤1,  = 2, or ≥3). To test for the association between the category of copy number for each probe and disease status, we used logistic regression with adjustments for age and sex. Odds ratios (ORs) and their 95% confidence intervals (CIs) were calculated. We also calculated the concordance rate of copy number category across the four probes. The Cochran-Armitage trend test was used to find the linear relationship between the copy number of target probes and GC risk. Student’s t and Mann-Whitney U (if not normally distributed) were used to compare the copy number of each probe between GC patients and healthy controls. A two-tailed p value<0.05 was considered statistically significant. Statistical analyses were performed by JMP software version 9.0 (SAS Institute Inc., NC, USA).

## Results

### Study subjects

**Figure 1:**
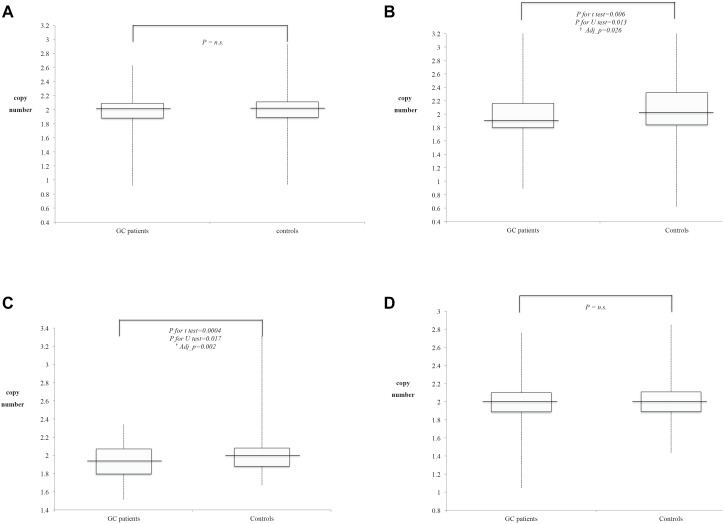
Distribution of individuals’ non-integer copy number on four probes of chromosome 5q22 (110 GC patients and 325 controls). A. *APC_intron8*; B. *APC_exon9*; C. *SRP19*; D: *REEP5*. *Line represents the median value of the non-integer copy number on GC groups and healthy groups, ^†^Adj_p: Adjustment for age and sex.

All 110 GC patients and 325 control subjects had copy number information for at least one of the four probes at 5q22. The distribution of copy number values for each probe is shown in **Figure 1**. GC patients were significantly older than the healthy controls (age; mean±SD: GC patients = 66.5±13.8, Controls = 62.5±9.7; p = 0.001). Men accounted for a larger proportion (61.8%) of GC patients, but they comprised only 46.4% of the healthy controls (p = 0.005). Among the GC patients, 55 had *H. pylori* infection, 28 were not infected by *H. pylori,* and 27 had no such information. 37 of GC patients had histologic Lauren’s classification (19 diffuse, 16 intestinal, and 2 mixed subtypes); 34 had differentiation grade (3 well-differentiated, 9 moderately-differentiated and 22 poorly-differentiated); 45 had AJCC tumor stage (12 stage I, 7 stage II, 13 stage III, and 13 stage IV).

### Association between CNV and gastric cancer

As expected, most of the participants in the study had 2 copies of the nearby CNV segment: ranging from 78.2 to 92.6% among the 4 probes in the controls and from 87.3 to 93.6% in the GC patients. The concordant rate for the copy number across the 4 probes ranged from 80.5 to 93.1% (**Table S3 in File S1**). This copy number was frequently variable in a larger region of known functional *APC* and *SRP19*. Therefore, the variations might account for the distance length and functional variations (**Table S3 in File S1**). For the probe of *APC-exon9*, 9.1% of the GC patients belonged to copy number category 3, whereas 17.5% of the controls were in category 3 (OR = 0.48, 95% CI: 0.22–0.93; crude p = 0.04, age/sex-adjusted p = 0.07, **Table 1**). Similarly, fewer GC patients were in category 3 compared to the control subjects for the other three probes, but the differences were not significant (**Table 1**). Furthermore, a dose-dependent relationship was observed between GC and the copy number of the probe for *APC-exon9* (**Table 1**). This implies that the controls tended to have a higher proportion of gain of copy numbers than cases with a trend p value of 0.026 (age/sex adjusted p = 0.067 for the trend test).

**Table 1:** The association between the copy number category between GC patients (n = 110) and healthy controls (n = 325).

Genes	Copy Number	GCs	Controls	Crude	Adjusted
		n (%)	n (%)	P value	P value§
***APC-intron8***	Cont.†	1.99/0.21 (0.92, 2.63)	1.99/0.22 (0.93, 2.93)	0.48	0.17
	≤1	4 (3.6)	6 (1.9)	0.29	0.40
	2‡	104 (94.6)	308 (94.8)	reference	reference
	≥3	2 (1.8)	11 (3.3)	0.53	0.38
				(0.19)	(0.23)
***APC-exon9***	Cont.†	1.99/0.36 (0.89, 3.48)	2.05/0.48 (0.62, 6.59)	0.01	0.03
	≤1	4 (3.6)	7 (2.2)	0.50	0.59
	2‡	96 (87.3)	261 (80.3)	reference	reference
	≥3	10 (9.1)	57 (17.5)	0.04	0.07
				(0.03)	(0.07)
***SRP19***	Cont.†	1.94/0.27 (1.51, 2.34)	1.97/0.20 (1.67, 3.31)	0.02	0.002
	≤1	0 (0.0)	0 (0.0)	N/A*	N/A*
	2‡	110 (100.0)	310 (95.4)	reference	reference
	≥3	0 (0.0)	15 (4.6)	0.02	N/A
				(N/A)*	(N/A)*
***REEP5***	Cont.†	2.00/0.21 (1.04, 2.76)	1.98/0.22 (1.43, 2.85)	0.59	0.75
	≤1	3 (2.8)	0 (0.0)	0.02	N/A*
	2‡	103 (93.6)	312 (96.3)	reference	reference
	≥3	4 (3.6)	12 (3.7)	1	1
				(0.23)	(0.31)

*N/A: not available.

† The continuous copies were reported by Median/Interquartile Range (IQR) (min, max) and the p values were calculated by nonparametric Mann-Whitney U test.

‡ The category copies were reported by n (%) and the p values were calculated by Chi-square/Fisher’s exact test and Cochran-Armitage trend test (brackets).

§ The p values were calculated by multivariate logistic regression with adjustments for age and sex.

Because the copy number was estimated from the real-time PCR data, the original copy number was not an integer and not normally distributed. Therefore, we also test the nonparametric association between the non-integer data on the copy number and the disease status. The medians and interquartile ranges (IQRs) of the copy number of each probe (*APC-intron8, APC-exon9, SRP19,* and *REEP5*) were compared between GC patients and healthy controls (**Table 1**). Similar to the results from the integer copy number, the GC patients had a significantly lower copy number compared to the controls for the *APC-exon9* (crude p for t test = 0.006, crude p for Mann-Whitney U test = 0.013, sex/age-adjusted p = 0.026) and *SRP19* (crude p for t test = 0.0004, crude p for Mann-Whitney U test = 0.017, sex/age-adjusted p = 0.002) probes (**Figure 1B** **and** **Figure 1C**). There was no significant difference for the other two CNV probes (*APC-intron8* and *REEP5*) (**Figure 1A** **and** **Figure 1D**). Further analysis of the correlations between copy number and GC clinical pathological classifications, the results showed that no significant copy number difference at exon-9 of *APC* gene regardless of histological, differentiation grades or TNM stage, that might due to small sample sizes (**Table 2**).

**Table 2:** Correlation between *APC-exon9* copy number variations and clinic pathological of gastric cancer.

		APC-exon9 copy number					
	N	Cont.†	P value†	CN≤1‡	CN = 2‡	CN≥3‡	P value‡
		Median/IQR		n (%)	n (%)	n (%)	
***H. pylori***							
yes	55	2.02/0.38	0.43	2 (3.6%)	45 (81.8%)	8 (14.6%)	0.77
no	28	2.04/0.44		1 (3.6%)	25 (89.3%)	2 (7.14%)	
**Lauren Classification**							
Intestinal	16	2.04/0.37	0.77	0 (0%)	15 (93.8%)	1 (6.3%)	1.00
Diffuse	19	1.92/0.42		1 (5.3%)	16 (84.2%)	2 (10.5%)	
Mixed	2	1.93/0.13		0 (0%)	2 (100%)	0 (0%)	
**Differentiation grade** *							
WD	3	2.11/0.16	0.21	0 (0.0%)	3 (100.0%)	0 (0.0%)	1.00
MD	9	1.99/0.49		0 (0.0%)	9 (100.0%)	0 (0.0%)	
PD	22	1.87/0.38		2 (9.1%)	18 (81.8%)	2 (9.1%)	
**TNM stage**							
I	12	1.93/0.54	0.83	1 (8.3%)	11 (91.7%)	0 (0.0%)	0.93
II	7	1.92/0.46		0 (0%)	6 (85.7%)	1 (14.3%)	
III	13	1.87/0.49		0 (0%)	12 (92.3%)	1 (7.7%)	
IV	13	1.98/0.33		1 (7.7%)	11 (84.6%)	1 (7.7%)	

*WD, well differentiated; MD, moderately differentiated; PD, poorly differentiated.

† The continuous copies were reported by Median/Interquartile Range (IQR) (min, max) and the p values were calculated by nonparametric Mann-Whitney U/Kruskal-Wallis test.

‡ The category copies were reported by n (%) and the p values were calculated by Chi-square/Fisher’s exact test.

## Discussion

This study used 4 probes to investigate the association between copy number variation at chromosome 5q22 and GC. This region covers three genes: the 3′ end of the *APC* gene, the entire *SRP19* gene, and the 3′ of the *REEP5* gene. For the probe of *APC-exon9*, the control had higher copy number values than the GC patients in all three analyses (i.e., copy number category, trend test, and non-integer copy number). The probe of *SRP19* also had a significantly higher copy number (based on copy number category and non-integer analyses) in the controls than in GC patients. For the *APC-intron8* and *REEP5* probes, the copy number values were not significantly different between GC patients and the control group in any of the three analyses. The results of this study indicate that a reduced copy number in the region of this CNV may be associated with a higher risk of gastric cancer.

The *APC* gene containing 15 exons is located at chromosome 5q21–22. Most mutations of the *APC* gene were observed in exon 15 in FAP patients [26] and GC patients [29]. Six mutations in the alternatively-spliced region of exon 9 have been documented to be associated with FAP [26] and colon cancer [27], but these mutations have not been reported to be associated with GC. This study is the first to report the association between the copy number at *APC-exon9* and GC. A possible Wnt/β catenin/Tcf signaling transduction pathway associated with APC has been reported for GC [30]. One main function of APC is thought to regulate free β catenin and so loss of APC function may result in the instability of β catenin complex and the cellular accumulation of β catenin. Upon translocation to the nucleus, β catenin serves as an activator of T cell factor-dependent transcription, leading to an increased expression of several specific target genes that may be involved in the occurrence of gastric lesions ranging from chronic gastritis, gastric atrophy, intestinal metaplasia, dysplasia to finally gastric adenocarcinoma [31].

The CGH platform has been widely used for cancer studies, and the probes of this platform often cover a DNA region of longer than 1 kb DNA region. Therefore, the CGH approach is limited in pinpointing CNV segments when the changes are less than 1 kb. In this study, we used specific TaqMan probes labeled by different reporter fluorophores (VIC and FAM) in a single reaction. This approach allowed us to detect a more subtle DNA change. PCR amplification for each tested probe based on ABI TaqMan have been evaluated to be nearly 100% efficient [32]. Recently, this method has been widely used for other diseases, such as age-related macular degeneration and allergic asthma [33], [34]. Before copy number genotyping, our genomic DNA concentrations were rigorously quantified and controlled using two independent methods: UV absorbance (rational ratio range of OD 260/280: 1.8±0.2) and PCR amplification of *RPPH1* (rational Ct range of VIC: 25–27). Amplification of *RPPH1* was performed at the same well as the probe to protect against artificial variations (such as differences in DNA loading or erroneous detection of the null genotype). Therefore, our method can be considered to be reliable for quantitative characterization of the fragmental CNV. Based on the previous CGH experiments, studies have reported that 2% of loss copies and 2% of gain copies at 5q22 in healthy French men [25]. We used TaqMan probes, which have a higher resolution to detect a smaller region of copy number variation, and found that the percentage of loss copies in the 4 probes ranged from 0 to 2.7% in the healthy Taiwanese. However, a higher proportion of gain copies ranging from 2.7% (*REEP5*) to 14.1% (*APC-exon9*), were observed in the healthy men. Similar to the data for male participants, the frequency of gain copies of *APC-exon9* was also high for female participants (20.2% of gain copies). However, another study investigating the Japanese population also reported a higher proportion (20.6%) of gain copy number at 5q where our investigated CNV is located [9].

As expected, most participants had 2 copies of the CNV segment among the four probes in all subjects. Only an 80% concordant rate was observed between copy numbers measured by the *APC-exon9* probe and the *SRP19* probe (**Table S3 in File S1**). This is actually the lowest concordance rate of all the pairwise rates between the probes, nevertheless, both probes were significantly associated with GC. It is possible that variation 7468 is not a continuous CNV, but it is regarded so because it was identified using array CGH, a technique with a lower resolution than those available today (i.e., it might consist of several shorter regions with variable copy numbers). Therefore, the variable boundary of the gene might be narrowed down from *APC-exon9 to SRP19*. As far as the front of *APC-exon9* is concerned, whether other variable regions associated with GC exist requires further exploration. In this study, there were no significant differences in copy numbers between GC patients and controls in *APC-intron8* and even *APC-intron7* (data not shown).

As is the case with most research efforts, our study had limitations. The sample size used in this study might not have been sufficient to detect a CNV with a small effect. Furthermore, we have limited information on clinic pathological characteristics (such as *H. pylori* infection, histologic grade, differentiation grade and tumor stage making it difficult for testing interaction of CNV and these parameters. More subjects were needed to confirm this result in the future. In conclusion, losses of a CNV at 5q22 (Variation 7468), especially in the DNA region surrounding APC-exon 9, may be associated with a higher risk of gastric cancer. A loss of this CNV may serve as a novel biomarker to identify high-risk individuals. Nevertheless, a large association-study is warranted to confirm the usefulness of this biomarker and the detailed mechanism remains to be clarified.

## Supporting Information

File S1 **Combined Supporting** Information file. Table S1 in File S1. Genetic abnormality at chromosome 5q22 in GC studies. Table S2 in File S1. Information from four probes at chromosome 5q22 and internal probe at chromosome 14q11. Table S3 in File S1. The concordance of copy number between each adjacent probe (110 GC patients and 325 healthy controls). Figure S1 in File S1. The locations of the four probes at the CNV containing *APC/SRP19/REEP5* genes.
(DOCX)
