# Supplementary material for: The Association between DNA Copy Number Aberrations at Chromosome 5q22 and Gastric Cancer
Source: PLoS One. 2014 Sep 11;9(9):e106624. doi: 10.1371/journal.pone.0106624 (PMC4161348; doi:10.1371/journal.pone.0106624)
Supplement: File S1 — Combined Supporting Information file. Table S1 in File S1. Genetic abnormality at chromosome 5q22 in GC studies. Table S2 in File S1. Information from four probes at chromosome 5q22 and internal probe at chromosome 14q11. Table S3 in File S1. The concordance of copy number between each adjacent probe (110 GC patients and 325 healthy controls). Figure S1 in File S1. The locations of the four probes at the CNV containing APC/SRP19/REEP5 genes. (DOCX) [file pone.0106624.s001.docx]

**Supplementary Data**

**Table S1. Genetic abnormality at chromosome 5q22 in GC studies**

| **References** | **Subjects** | **Methods^†^** | **Chromosome** | | **Results**  **(the association of 5q variation with GC)** |
| --- | --- | --- | --- | --- | --- |
|  |  |  | **Gains** | **Losses** |  |
| Wu, 2001, Taiwan | 53 GC patients | CGH | 8q21-24  6q16-22  11q12-23  13q21-23  7p12-15  17q12-21  20q11-13 | 16q21-24  19p  5q21-22 (19%)  1p32-33  3p14-16  4q | Copies loss at 5q21-22 were higher in intestinal GC than in diffuse GC (32% vs. 4%, p<0.05) |
| Wu, 2002, Taiwan | 62 gastric adenocarcinomas patients and 6 GC cell lines | CGH | 17q24-qter  20q13-qter  1p32-36  22q12-qter  17p13-pter  16p13-pter  6p21-pter  20p12-pter  7p21-pter  3q28-qter  13q13-q14 | 18q-qter  3p12  3p25-pter  5q14-q23 (8%)  9p21-p23 | 1. Copies loss at 5q14-23 were associated with GC of advanced TNM stages (p=0.015) and liver metastasis (p=0.03)  2. MSH3, CTNNAI, APC and MCC on 5q had been shown to be abnormal in GC |
| Kimura, 2004, Japan | 102 primary GC patients | CGH | 20q, 8q  20p, 7q  17q, 5p  13q | 19p, 18q  5q (27%)  21q, 4p  4q, 15q  17p | Copies loss at 5q were not association with cancer-stoma relationship, TMN category, lymphatic and venous invasion, and pathological stage. |
| Morohara, 2005, Japan | 34 primary GC patients | CGH | 5p  5q  7q  10p | 16q  22q | Significant differences were found in the rate of gains of 5q between primary tumors with and without peritoneal metastasis and peritoneal cytology (p=0.007). |
| Nishimura, 2008, Japan | 707 GC patients | CGH | 20q13 (38.9%)  8q23 (31.7%)  17q21 (20.5%)  13q21 (19.0%)  7p14 (17.0%)  11q13 (16.7%)  1q25 (12.2%)  3q26-q28 (11.9%)  5p13 (10.6%) | 19p13 (20.9%)  17p12 (17.7%)  18q22 (16.1%)  5q14-q21 (15.4%)  4q32 (13.7%)  16p13 (13.4%)  9p21 (12.0%)  22q12 (11.2%)  1p36 (10.2%) | Gene mutations at 5q14-q21 were frequently observed in GC patients. |
| Uchida, 2010, Japan | 20 gastric cancer  20 adenomas | aCGH | ***CIS****  8q, 20q  ***HGA***  8q, 7pq  ***LGA***  7q21.3-q22.1 | ***CIS****  5q (50%)  17p  ***LGA***  5q (43%) | The most frequent aberrations in gastric cancer were losses at 5q (50%) and 17p (50%), and the most frequent LGA were also loss at 5q (43%). |
| Hong, 2010, Korea | 145 gastric cancer patients | Microsatellites | ***-*** | 3p, 4p, 5q, 8p, 9p, 13q, 17p and 18q | 35% of 145 GC patients had chromosome 5q loss. Chromosomal 5q loss were concurrently related with the intestinal- or mixed-type histology |
| Van Dekken, 1999, The Netherland | 28 gastroesophageal junction adenocarcinomas | CGH | 5p14, 6p12-21.1  7p12, 8q23-24.1  12q21.1, 15q25  17q12-21 19q13.1  20p12  20q12-13  Xq25 | 3p14  5q14-21  9p21  14q31-32.1  16q23  18q21  21q21 | Minimal regions of overlap for deletions were assigned to 5q14-21 (APC, MCC). |
| Buffart, 2007, The Netherlands | 11 intestinal-type  10 pyloric gland adenomas | aCGH | ***Intestinal-type***  11q23.3  9q33.1-34.13  8q24.11  ***Pyloric gland***  20 | ***Intestinal-type***  13q21.1-q31.3  6p21.1-q16.1  5q22.1-q23.2  10  ***Pyloric gland***  5q21.1, 6q | The 5q21.1-23.2 location of chromosome 5 was frequent aberrations in intestinal-type gastric adenoma and also in pyloric gland gastric adenomas. |
| Vauhkonen, 2005, Finland | 25 intestinal-type  12 diffuse-type | Microsatellites | - | 5q23.2  5q33.1  18q21.33  21q21.1 | The markers detected LOH at chromosomal arms 5q, 18q, and 21q with a sensitivity equal to markers used in previous studies. |
| Gümüs-Akay 2009,Turkey | 43 gastric adenocarcinomas patients | HR-CGH | 7q, 8q  7p, 1q  13q, 20q | 18q  5q (21%)  14q | The most common losses were found on arms 18q (26%), 5q (21%), and 14q (21%). |

* CIS: gastric carcinoma in situ; HGA: high-grade adenomas; LGA: low-grade adenomas

† CGH: Comparative genomic hybridization; aCGH: array CGH; HR-CGH: high-resolution-comparative genomic hybridization

**Table S2. Information from four probes at chromosome 5q22 and internal probe at chromosome 14q11**

| **Probes** | **Genes Name** | **Genes**  **Length (kb)** | **Assays ID** | **Assay locations**  **(NCBI build 36)** |
| --- | --- | --- | --- | --- |
| ***APC-intron8*** | *Adenomatous polyposis coli* | 138.718 | Hs03565458 | Chr5: 112,150,103 |
| ***APC-exon9*** |  |  | Hs03572291 | Chr5: 112,156,139 |
| ***SRP19*** | Signal recognition particle 19kDa | 6.611 | Hs03557421 | Chr5: 112,225,109 |
| ***REEP5*** | Receptor accessory protein 5 | 45.950 | Hs02998972 | Chr5: 112,241,880 |
| ***RPPH1*** | *Ribonuclease P RNA component H1* | 0.340 | HS03297761-s1 | Chr14: 20,811,230 |

**Table S3. The concordance of copy number between each adjacent probe (110 GC patients and 325 healthy controls)**

| **Regions** | **Length (bp)** | **Number of concordant copies (rate; %)** | | |
| --- | --- | --- | --- | --- |
|  |  | **Total** | **GC patients** | **Healthy controls** |
| ***APC-intron8***  ***/APC-exon9*** | 6,036 | 365 (83.9) | 100 (90.9) | 265 (81.5) |
| ***APC-exon9***  ***/SRP19*** | 68,970 | 350 (80.5) | 96 (87.3) | 254 (78.2) |
| ***SRP19***  ***/REEP5*** | 16,771 | 404 (93.1) | 103 (93.6) | 301 (92.6) |

**Figure S1. The locations of the four probes at the CNV containing *APC/SRP19 /REEP5* genes**

**
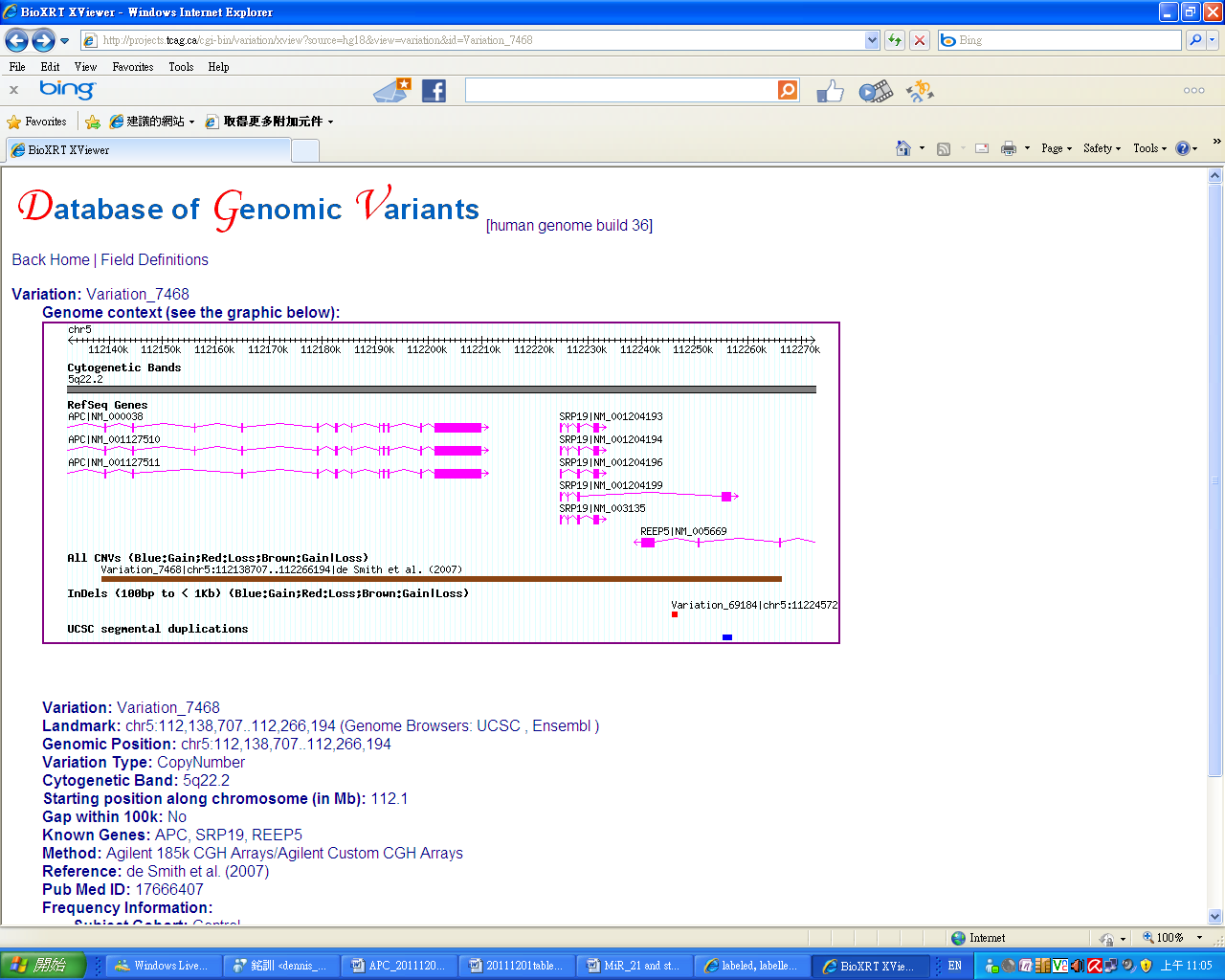
**

* Arrowheads represented the specific probes in this study
